# Post-transplant donor-specific anti-HLA antibodies with a higher mean fluorescence intensity are associated with graft fibrosis in pediatric living donor liver transplantation

**DOI:** 10.3389/fped.2023.1172516

**Published:** 2023-04-25

**Authors:** Ryoichi Goto, Yasutomo Fukasaku, Yoshikazu Ganchiku, Norio Kawamura, Masaaki Watanabe, Takuji Ota, Kanako C. Hatanaka, Tomomi Suzuki, Tsuyoshi Shimamura, Akinobu Taketomi

**Affiliations:** ^1^ Department of Gastroenterological Surgery I, Hokkaido University Graduate School of Medicine, Sapporo, Japan; ^2^ Department of Transplant Surgery, Hokkaido University Graduate School of Medicine, Sapporo, Japan; ^3^ Center for Development of Advanced Diagnostics, Hokkaido University Hospital, Sapporo, Japan; ^4^ Division of Organ Transplantation, Hokkaido University Hospital, Sapporo, Japan

**Keywords:** LDLT, living donor liver transplantation, donor specific antibodies, graft fibrosis, graft age, FIB4 index

## Abstract

The roles of post-transplant anti-HLA donor specific antibody (DSA) in pediatric liver transplantation (LT), including therapeutic strategies, remain controversial. This study aimed to identify the risks of post-transplant DSA for graft fibrosis progression in pediatric living donor LT (LDLT). We retrospectively evaluated 88 LDLT pediatric cases between December 1995 and November 2019. DSAs were assessed with single antigen bead test. Graft fibrosis was histopathologically scored with METAVIR and the centrilobular sinusoidal fibrosis system. Post-transplant DSAs were detected in 37 (52.9%) cases at 10.8 (1.3–26.9) years post-LDLT. The histopathological examination of 32 pediatric cases with post-transplant DSA revealed that 7 (21.9%) with a high DSA-MFI (≥9,378) showed graft fibrosis progression (≥F2). No graft fibrosis was observed in the subjects with a low DSA-MFI. The risk factors for developing graft fibrosis in pediatric cases with post-transplant DSA were an older graft age (>46.5 years old), lower platelet count (<10.7 × 10^4^/ml) and higher Fib4 index (>0.7807, recipient age; >1.8952, donor age). Limited efficacy of additional immunosuppressants was observed in DSA positive pediatric cases. In conclusion, pediatric cases with a high DSA-MFI and risk factors should undergo a histological examination. The appropriate treatment for post-transplant DSA in pediatric LT needs to be determined.

## Introduction

The recent development of an assay using single antigen beads (SABs) for anti-HLA antibodies has facilitated clinical studies of organ transplantation (1, 2). Post-transplant anti-HLA donor specific antibody (DSA) has been recognized as a factor preceding graft failure in kidney transplantation (3). Recent studies have identified harmful DSAs in cases of kidney transplantation, including their classes, types and titers (4–6). Furthermore, complement-binding anti-HLA antibodies identified by a C1q-binding assay predicted antibody-mediated rejection in kidney transplantation (7). Consistently, post-transplant DSAs have a detrimental effect on the clinical outcome in liver transplantation (LT) (8–10). *De novo* DSA presence was shown to be an independent risk factor of liver graft fibrosis progression in LT recipients for hepatitis C virus (HCV)-related liver cirrhosis (11). In addition, *de novo* DSAs were reportedly associated with graft fibrosis progression in pediatric LT (9). A protocol biopsy in a pediatric LT case showed graft fibrosis progression in the presence of post-transplant DSAs (12).

However, liver grafts are considered highly resistant to antibody-mediated injury (13). Feng et al. showed that the presence of DSAs does not necessarily indicate unsuccessful operational tolerance in pediatric LT (14, 15). Thus, the roles of DSA in the development of liver graft fibrosis in pediatric LT remains unclear.

In the present study, we examined the role of post-transplant DSA in pediatric living donor LT (LDLT). In addition, we described our treatment strategies for subjects with post-transplant DSA.

## Patients and methods

### Patients

**Figure 1:**
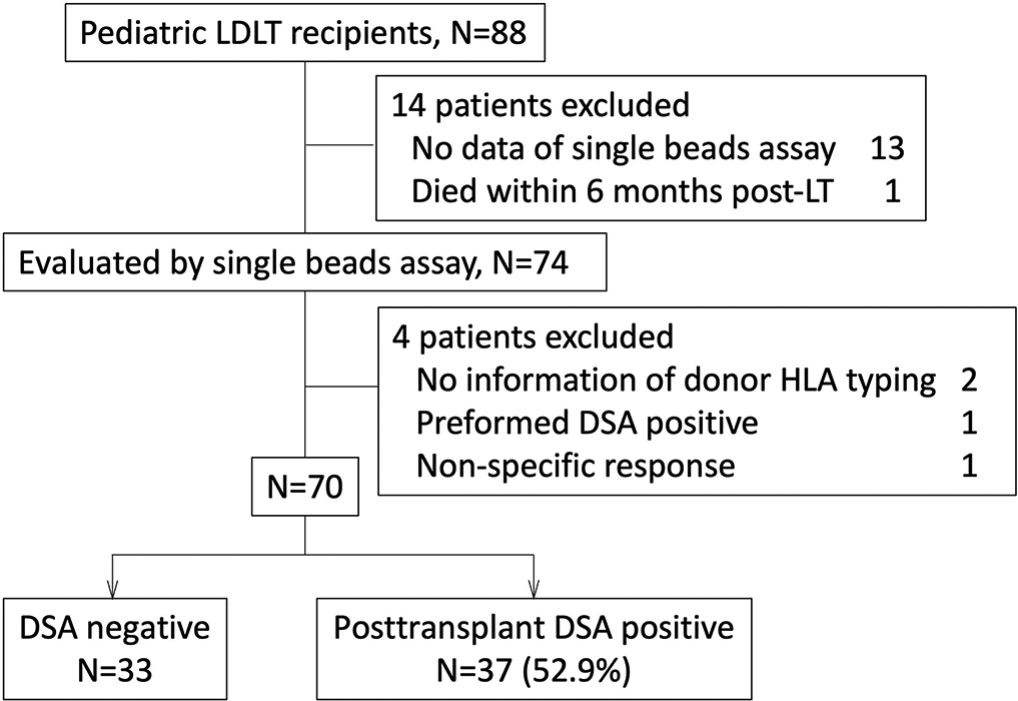
We enrolled pediatric patients who underwent LT or were followed up post-LT in our institute between December 1995 and November 2019. The patients who did not have any data evaluating by single beads assay (*n* = 13), or donor HLA typing (*n* = 2), and had the data with non-specific response (*n* = 1), preformed DSA (*n* = 1) were excluded from this study. A patient who died within 6 months post-LT was also excluded. Eventually 70 patients were enrolled in this study. DSA, donor specific anti-HLA antibody; LDLT, living donor liver transplantation.

The study protocol followed the ethical guidelines of the 1975 Declaration of Helsinki and was approved by the institutional review board at Hokkaido University Hospital (#017-0104). We retrospectively evaluated 88 pediatric recipients who underwent LDLT between December 1, 1995, and November 31, 2019, and were followed up in Hokkaido University Hospital (Figure 1).

The sera of 75 pediatric recipients were evaluated by an SAB assay using Labscreen® (One Lambda Inc., Canoga Park, CA, USA). The patients without donor HLA typing information (*n* = 2), with preformed DSA (*n* = 1), with difficulty undergoing an evaluation for non-specific response to an SAB assay (*n* = 1) and who died within 6 months post-LT (*n* = 1) were excluded from the study (Figure 1). The recipients who had no DSA diagnosed by SAB assays were defined as “DSA-negative” (Table 1). Rejection episodes were confirmed by a histological examination of liver biopsy samples.

**Table 1:** Characteristics of the pediatric LDLT recipients who developed post-LT DSA.

Variables	DSA-negative	Post-LT DSA-positive	*p*
	*N* = 33	*N* = 37	
Male, *n* (%)	9 (27.3%)	18 (48.7%)	0.067
Donor male, *n* (%)	13 (39.4%)	16 (43.2%)	0.74
Age of recipient, years	1.32 (0.5–15.6)	1.51 (0.5–17.0)	0.50
Age of donor, years	36.9 (22.0–60.0)	32.0 (23.7–66.0)	0.79
MELD or PELD	4.2 ± 11.4	12.1 ± 12.0	0.0196*
Relationship with donor, *n* (%)			
Mother	14 (46.7%)	15 (40.5%)	0.44
Father	12 (40.0%)	15 (40.5%)	
Grandmother	2 (6.7%)	5 (13.5%)	
Others	2 (7.1%)	2 (5.5%)	
Primary disease, *n* (%)			
Biliary atresia	28 (84.9)	27 (73.0)	0.46
Fulminant hepatitis	1 (3.0)	3 (8.1)	
Alagille	0	2 (5.4)	
Others	4 (12.1)	5 (13.5)	
GvSv (%)	78.5 ± 23.1	77.8 ± 24.8	0.74
Cold ischemia time (min)	77.6 ± 46.7	78.9 ± 56.6	0.99
IS off, *n* (%)	8 (24.2%)	12 (32.4%)	0.45
Graft age at SAB assay	47.6 (26.0–61.9)	46.4 (32.6–77.7)	0.93
AR until 1 year post-LT, *n* (%)	14 (43.8%)	25 (69.4%)	0.065
Total bilirubin (mg/dl)	0.76 ± 0.46	0.95 ± 0.53	0.077
Albumin (g/dl)	4.27 ± 0.45	4.18 ± 0.46	0.75
AST (IU/L)	32.4 ± 17.6	34.2 ± 26.5	0.82
ALT (IU/L)	27.3 ± 24.2	35.7 ± 45.6	0.33
Platelet (×10^4^/ml)	22.3 ± 8.0	18.6 ± 6.7	0.047*
Fib4-index	0.44 ± 0.29	0.60 ± 0.56	0.16
Fib4-index (donor)	1.64 ± 1.07	2.17 ± 2.38	0.60
APRI	0.54 ± 0.31	0.77 ± 0.92	0.37
ALBI	−2.93 ± 0.38	−2.80 ± 0.46	0.35

Mann–Whitney *U* test or Fisher's exact test. Data are shown as the mean ± standard deviation or median. ALT, alanine aminotransferase; AST, aspartate aminotransferase; APRI, AST-to-platelet ratio index; ALBI, albumin-bilirubin index; AR, allograft rejection; DSA, donor-specific anti-HLA antibody; Fib4, fibrosis 4; GvSv, graft volume/standard liver volume ratio; IS, immunosuppressants; MELD, model for end-stage liver disease; PELD, pediatric end-stage liver disease; LDLT, living donor liver transplantation; LT, liver transplantation; SAB, single antigen beads.

* *p* < 0.05.

### HLA typing and detection of anti-HLA antibodies

Before transplantation, all recipients and donors were basically typed for HLA-A, HLA-B, HLA-Cw, HLA-DQ and HLA-DR using LabType SSO® (One Lambda Inc.). All patients have been basically examined to detect donor-specific anti HLA-A, HLA-B, HLA-Cw, HLA-DR, and HLA-DQ antibodies using the LABScreen® SAB assay since 2019. Patients with abnormal findings on liver functional tests or suspected antibody-mediated rejection have been evaluated with an SAB assay since 2011. A normalized mean fluorescence intensity (MFI) greater than 1,000 was considered a positive result. Normalized DSA-MFI was represented as the DSA-MFI value calculated from the negative control beads and negative serum sample, according to the manufacture's protocol. The DSA-MFI cut-off of 1,000 for “positivity” was set according to the manufacturer's instructions and referenced from a previous study (16). The DSA-MFI was evaluated as the maximum and sum of MFI, according to previous studies (17–20).

### Immunosuppressant protocol

As induction therapy, Basiliximab was administered to patients who underwent LDLT from October 2003 to January 2019 in Hokkaido University Hospital, in conjunction with a triple-immunosuppressant regimen of tacrolimus (target trough 10–15 ng/ml for 1 month post-transplantation), mycophenolate mofetil (MMF; daily 10–30 mg/kg) and methylprednisolone (mPSL; 0.5 mg/kg daily with withdrawal weekly base). Tacrolimus-based regimens (with or without MMF or mPSL) were usually administered during the maintenance phase.

### Histopathological and immunohistochemical examinations

**Figure 2:**
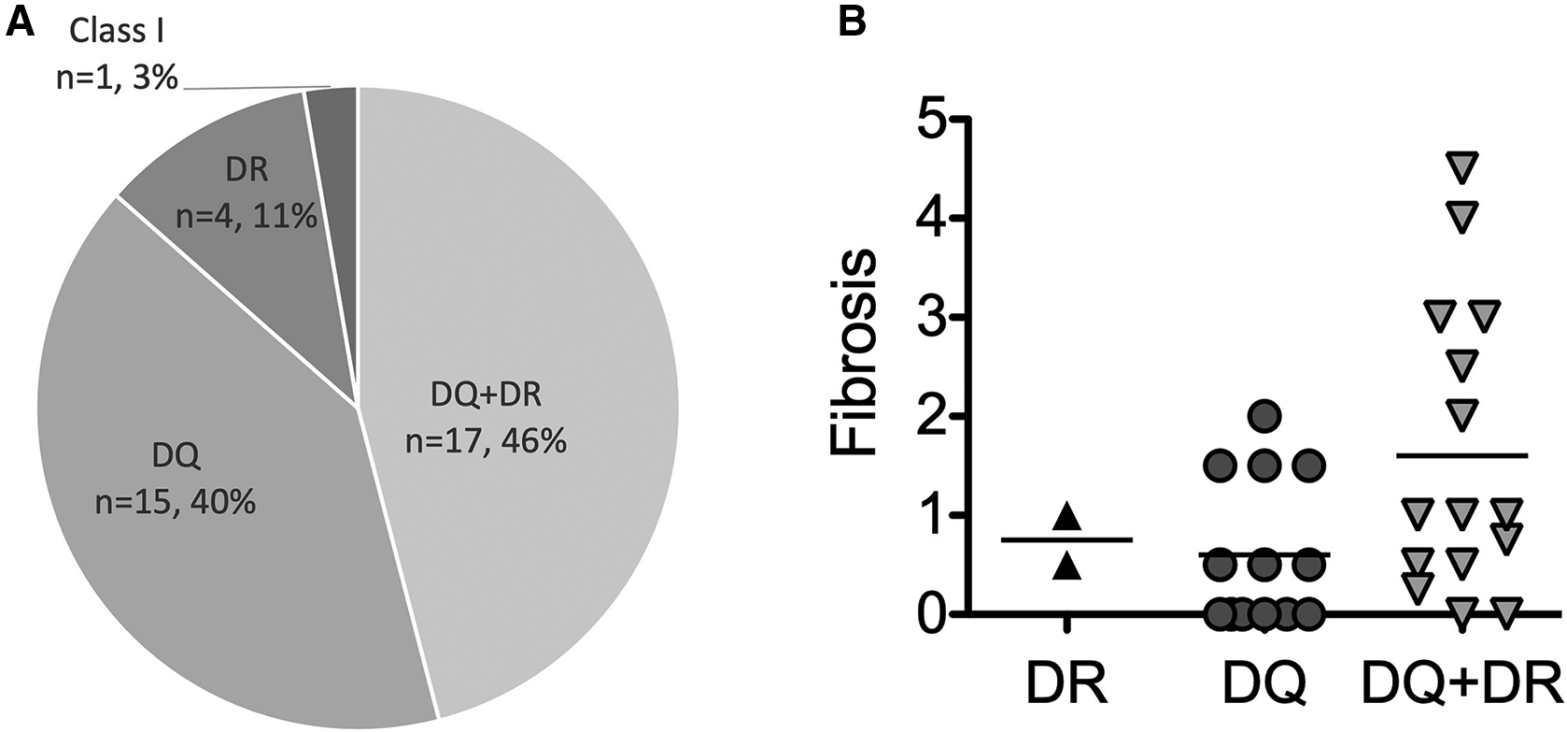
The type of post-transplant DSA. (**A**) The frequencies of types of post-transplant DSA among pediatric LT recipients (*n* = 37). (**B**) The correlation between the type of post-transplant DSA and graft fibrosis among the pediatric LT recipients who had available data histologically evaluated by liver biopsies (*n* = 32). The values of DSA-MFI and histological findings were collected at the time of the first detection of post-transplant DSA.

**Figure 3:**
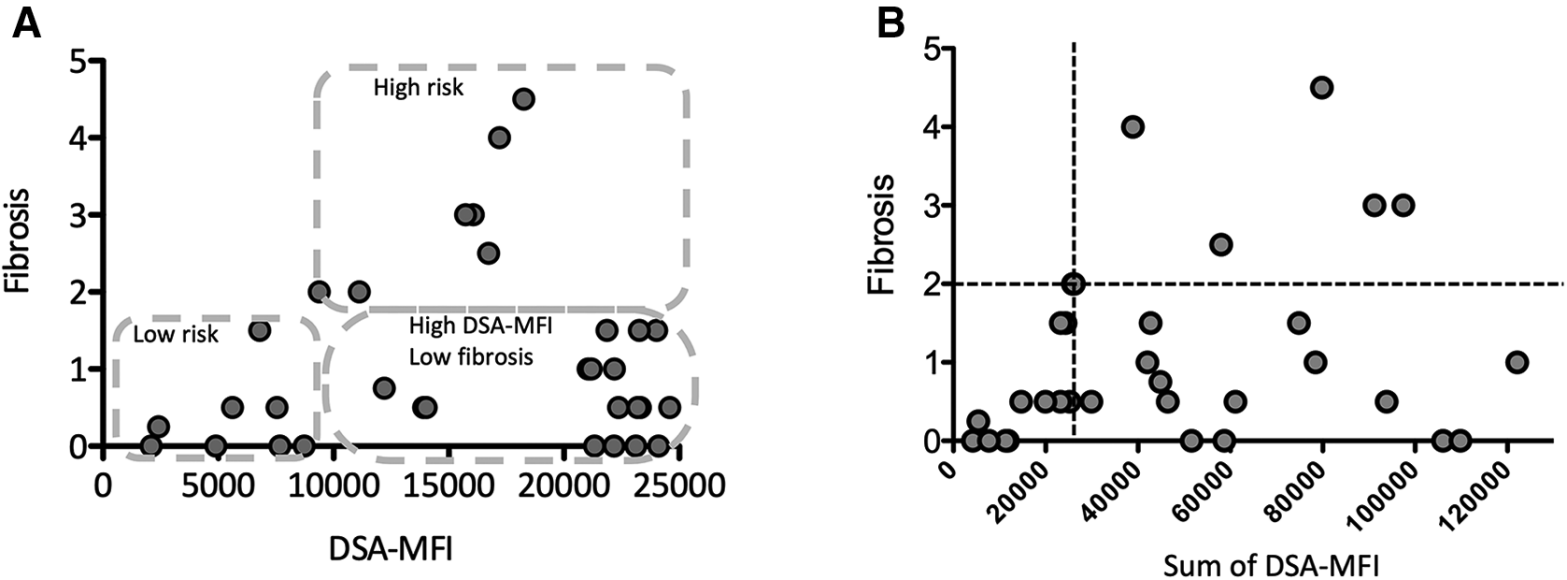
The correlation between the maximum (**A**) or sum of (**B**) the DSA-MFI and graft fibrosis. (**A**) The pediatric LT patients who developing post-transplant DSA were divided into 3 groups by the DSA-MFI cut-off value (9,300) and a fibrosis score of 2. (**B**) A graft fibrosis score of 2 (dotted line) and a DSA-MFI sum of 2,500 (dotted line) were used as cut-off values. The values of DSA-MFI and histological findings were collected at the time of the first detection of post-transplant DSA.

Histological findings were obtained from liver biopsy specimens using a 16-gauge cutting needle with a Pro-Mag ultra-automatic biopsy instrument (Argon medical device, inc., Frisco, Tx, USA). During the study periods, liver biopsy were performed in patients with abnormal liver functional test findings, those suspected of graft rejection, those with DSA positivity confirmed by an SAB assay and those in whom no histological evaluation had been conducted for several years despite receiving no immunosuppressant treatment. The histological data in Figures 2, 3 obtained by a liver biopsy were basically indicated for DSA positivity and collected around the same time as the SAB assays (11.6 ± 6.5 years post-LT). The histological scoring system was used for the METAVIR staging system (21), and the modified Dixon criteria were used for sinusoidal fibrosis around the central veins (22). C4d immunohistochemical staining of paraffin-embedded tissue blocks was performed using polyclonal human anti-C4d antibodies (BI-RC4D; Biomedica, Vienna, Austria). Linear to granular microvascular endothelial cell C4d staining was diagnosed as a positive result.

### Statistical analyses

**Figure 4:**
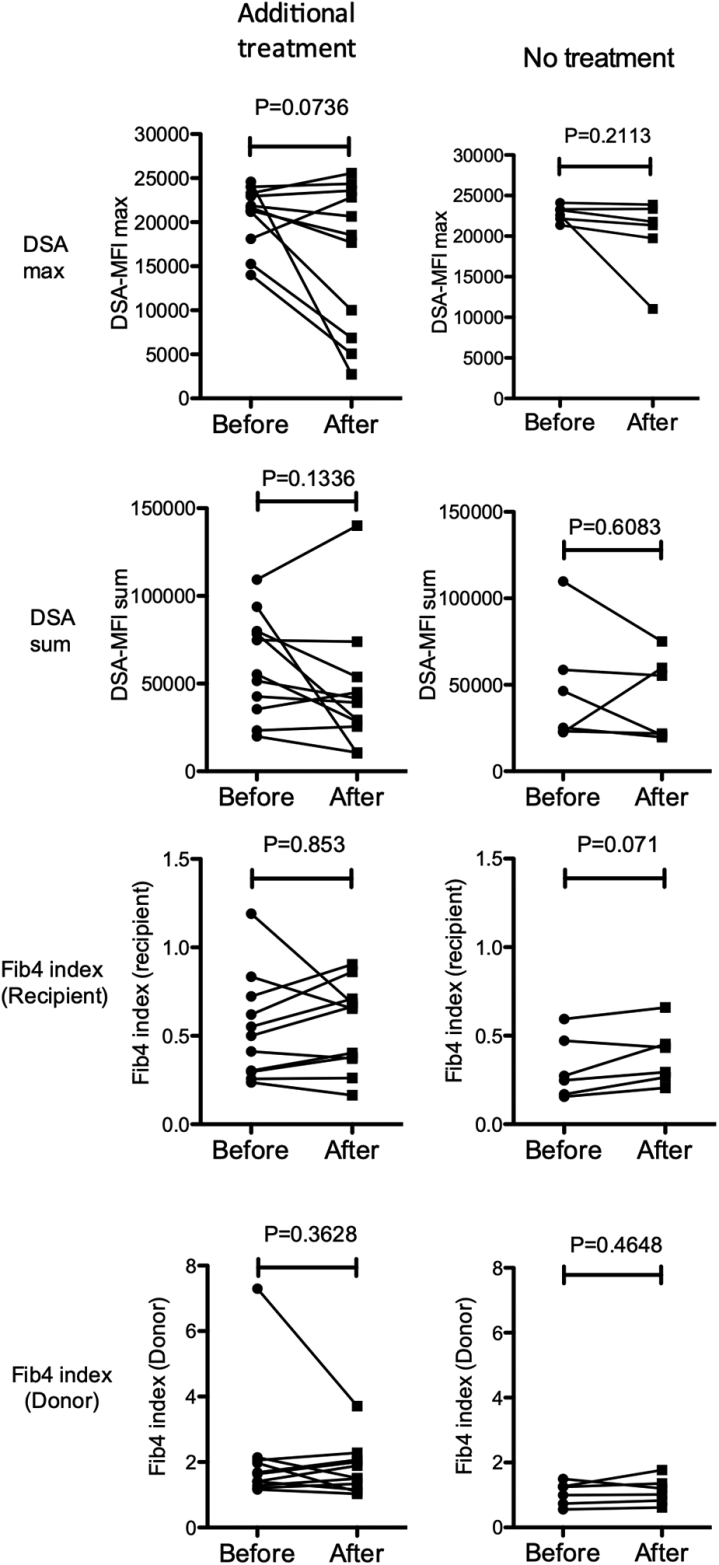
Changes in the maximum (top row) and sum (second row) of the DSA-MFI, Fib-4 indexes calculated by ages of the recipient (3rd row) and donor (4th row) after treatments with (left column, *n* = 11) or without (right column, *n* = 6) additional immunosuppressants for pediatric LT recipients with a high DSA-MFI and low fibrosis (*n* = 17). Paired *t*-test.

Patient characteristics were reported with median values and interquartile ranges (IQRs). The Mann-Whitney U test was used to compare continuous variables. Paired *t*-tests were used for evaluating treatment efficacies (Figure 4). The association between the frequencies of categorical variables was assessed by Fisher's exact test. A regression analysis was used to test the correlation with quantitative variables.

Statistical analyses were performed using the JMP Pro software program, version 16 (SAS institute Inc., Cary, NC, USA) and the GraphPad Prism software program, version 5 (San Diego, CA, USA). A *p*-value <0.05 was considered to indicate statistical significance in all cases.

## Results

### Post-transplant DSA positivity in pediatric LDLT patients

Of the 70 pediatric LT recipients enrolled in the current study (Figure 1), 37 (52.9%) showed post-transplant DSA positivity on SAB assays at 10.8 (1.3–26.9) years post-LT (Figure 1). The 37 pediatric patients with post-transplant DSA positivity had significantly higher MELD or PELD scores at LT (*p* = 0.0196) and lower platelet counts at the SAB evaluation (*p* = 0.047) than the patients without post-transplant DSA positivity (Table 1). In addition, patients with DSA positivity tended to be male (*p* = 0.067), have rejection episodes within 1 year post-LT (*p* = 0.065) and have a higher total bilirubin at SAB evaluation (*p* = 0.077) than those without DSA positivity (Table 1).

### Association of the DSA-MFI with graft fibrosis progression

Consistent with previous report (8), almost all post-transplant DSAs were class II HLA antibodies, i.e., antibodies against DQ + DR (*n* = 17, 46%), DQ (*n* = 15, 40%) and DR (*n* = 4, 11%) (Figure 2). Of interest, 6 of the 7 patients (86%) developing graft fibrosis ≥ F2 had post-transplant DSAs to both DQ and DR (Figure 2B). Furthermore, there was a correlation between a high DSA-MFI and advanced graft fibrosis (*r* = 0.65, Figure 3A). All patients developing graft fibrosis ≥ F2 had a high maximum DSA-MFI (>9,300) (Figure 3A) as well as a DSA-MFI sum exceeding 25,000 (Figure 3B). We also confirmed that no subjects with a low maximum DSA-MFI had a high DSA-MFI sum (Supplementary Figure S1). Importantly, patients with a high maximum DSA-MFI occasionally included those without graft fibrosis, suggesting that not all post-transplant DSAs were involved in graft fibrosis progression (Figure 3A).

We next categorized patients into 3 groups by cut-off values of graft fibrosis (score 2) and a DSA-MFI of 9,300, as shown in Figure 3A. On comparing these 3 groups, the graft age (*p* < 0.03), post-transplant DSAs against DQ7 (*p* = 0.012), platelet count (*p* = 0.036), Fib4 index calculated by recipient age (*p* = 0.042) and Fib4 index by donor age (*p* < 0.0043) were significantly different between the high-risk group and high-DSA-MFI, low-fibrosis group (Table 2). The cut-off values of continuous variables were 46.5 years old for the graft age, 10.7 × 10^4^/ml for the platelet, 0.7807 for the Fib4 index by recipient age and 1.8952 for the Fib4 index by donor age (Table 3).

**Table 2:** Risk factors for graft fibrosis progression in the presence of post-LT DSA.

Variables	Low risk	High DSA-MFI low Fibrosis	High risk	*p*
	*N* = 8	*N* = 17	*N* = 7	
Male, *n* (%)	5 (62.5%)	6 (35.3%)	4 (57.1%)	0.37
Recipient age at LT, years	2.1 (0.6–14.2)	1.3 (0.5–16.2)	2.2 (1.0–5.5)	0.36
Donor age at LT, years	32.3 (27.8–54.9)	31.4 (26.0–66.7)	44.0 (28.0–63.0)	0.081
Graft age at DSA detection	45.6 (39.3–69.7)	40.1 (33.3–68.6)	57.3 (46.5–77.7)*	<0.03*
Donor: Mother/Father/Grandparent, *n* (%)	4 (50%)/3 (38%)/1 (13%)	8 (47%)/8 (47%)/1 (6%)	1 (14%)/3 (43%)/3 (43%)	0.33
Primary disorder				
BA/Fulminant/Other, *n* (%)	7 (88%)/0/1 (13%)	12 (71%)/0/5 (29%)	4 (57%)/3 (43%)/0	0.098
GvSv (%)	56.4 ± 16.2	88.9 ± 23.6	77.9 ± 19.1	0.057
Graft volume (g)	289.2 ± 122.1	271.2 ± 48.8	276.0 ± 48.9	0.57
History of IS off, *n* (%)	3 (37.5%)	5 (29.4%)	3 (42.9%)	0.80
AR within 1 year, *n* (%)	4 (50.0%)	12 (70.6%)	5 (71.4%)	0.38
DSA: DQ + DR/DQ/DR, *n* (%)	4 (50%)/4 (50%)/0	4 (29%)/10 (59%)/2 (12%)	6 (86%)/1 (14%)/0	0.12
Include DQ7, *n* (%)	2 (25%)	4 (23.5%)	6 (85.7%)*	0.012*
Total MFI, *n* (%)	13,081.7 ± 7,988.8	60,534.1 ± 32,102.0*	59,639.8 ± 30,349.4*	<0.002 vs. Lo risk*
Years post-LT until SAB	10.2 (7.3–20.8)	7.9 (1.3–23.4)	12.5 (3.2–26.9)	0.15
Laboratory data at LBx				
Total bilirubin (mg/dl)	1.1 ± 0.7	0.8 ± 0.5	1.1 ± 0.5	0.40
Albumin (mg/dl)	4.3 ± 0.7	4.1 ± 0.4	4.1 ± 0.5	0.071
AST (IU/L)	25.6 ± 6.1	39.1 ± 37.7	35.9 ± 14.5	0.10
ALT (IU/L)	26.4 ± 22.6	45.2 ± 64.1	26.3 ± 14.7	0.29
Platelet (×10^4^/ml)	20.6 ± 6.4	19.3 ± 4.5	13.3 ± 6.2*	0.036 vs. Hi DSA lo Fib*
APRI	0.40 ± 0.21	0.81 ± 1.20	1.18 ± 0.83*	0.018 vs. Lo risk*
ALBI	−2.89 ± 0.72	−2.79 ± 0.33	−2.64 ± 0.47	0.093
Fib4 index (recipient age)	0.46 ± 0.15	0.42 ± 0.25	1.12 ± 1.09*	0.042 vs. Hi DSA lo Fib*
Fib4 index (donor age)	1.26 ± 0.61	1.64 ± 1.50	4.64 ± 4.17*	<0.0043 vs. Others*

Mann–Whitney *U* test or Fisher's exact test. Data are shown as the mean ± standard deviation or median. ALT, alanine aminotransferase; AST, aspartate aminotransferase; APRI, AST-to-platelet ratio index; ALBI, albumin-bilirubin index; AR, allograft rejection; DSA, donor-specific anti-HLA antibody; Fib4, fibrosis 4; GvSv, graft volume/standard liver volume ratio; IS, immunosuppressants; LT, liver transplantation; MFI, mean fluorescence intensity; SAB, single antigen beads.

* *p* < 0.05.

**Table 3:** Cut-off values of continuous variables for graft fibrosis progression.

Variables	Cut-off	AUC
Graft age	46.5	0.878
Platelet count (×10^4^/ml)	10.7	0.901
Fib4 index by recipient age	0.7807	0.833
Fib4 index by donor age	1.8952	0.955

AUC, area under the curve.

### Treatment for high-risk subjects with post-transplant DSA

**Figure 5:**
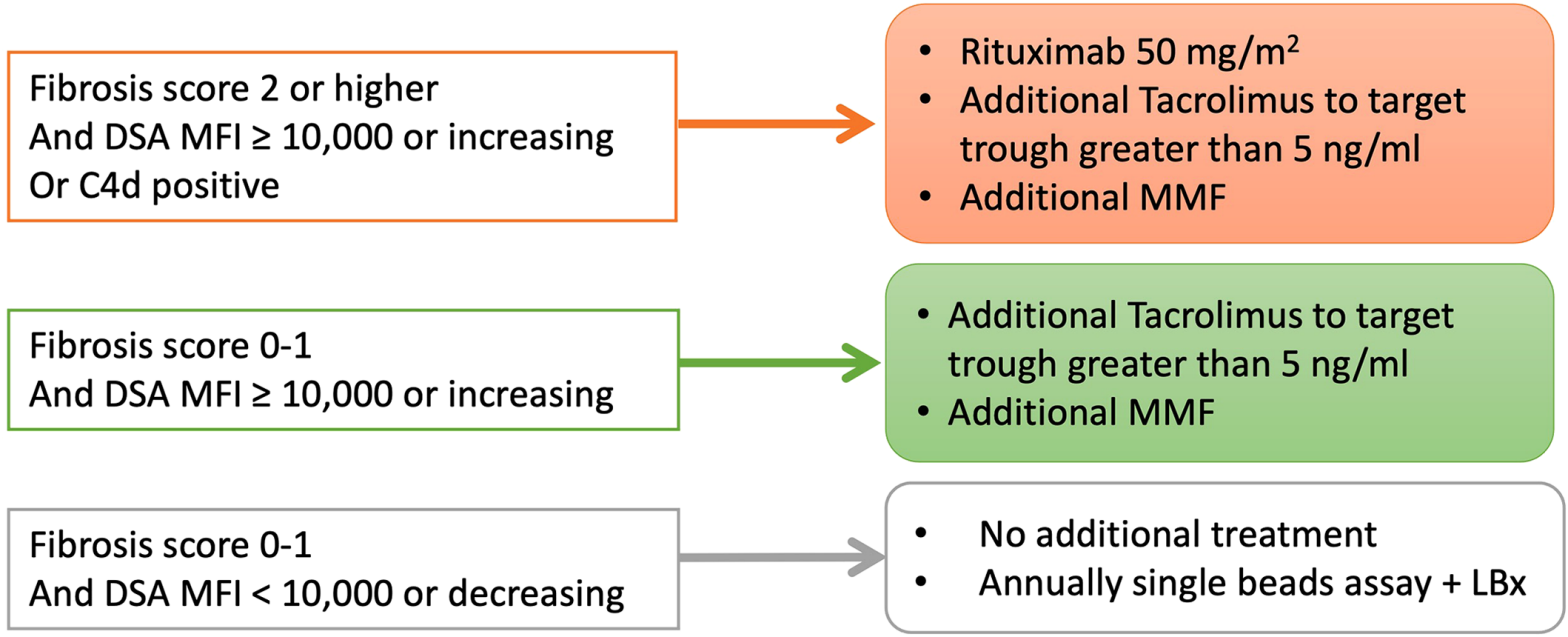
The treatment strategies for pediatric LT patients with post-transplant DSA in our institute since 2015. LBx, liver biopsy.

Although the best practice for post-transplant DSA positivity in LT is unclear, we have applied a treatment algorithm to patients with post-transplant DSA positivity since 2015 (Figure 5). Patients with both a DSA-MFI ≥ 10,000 and histological findings of graft fibrosis ≥2 were treated with 50 mg/kg of rituximab and tacrolimus, targeting a trough level of ≥5 ng/ml and MMF. Patients with a DSA-MFI ≥ 10,000 but no graft fibrosis progression (0–1) were basically treated with tacrolimus targeting a trough level of ≥5 ng/ml and additional MMF. For all other cases, no additional treatments were delivered.

**Figure 6:**
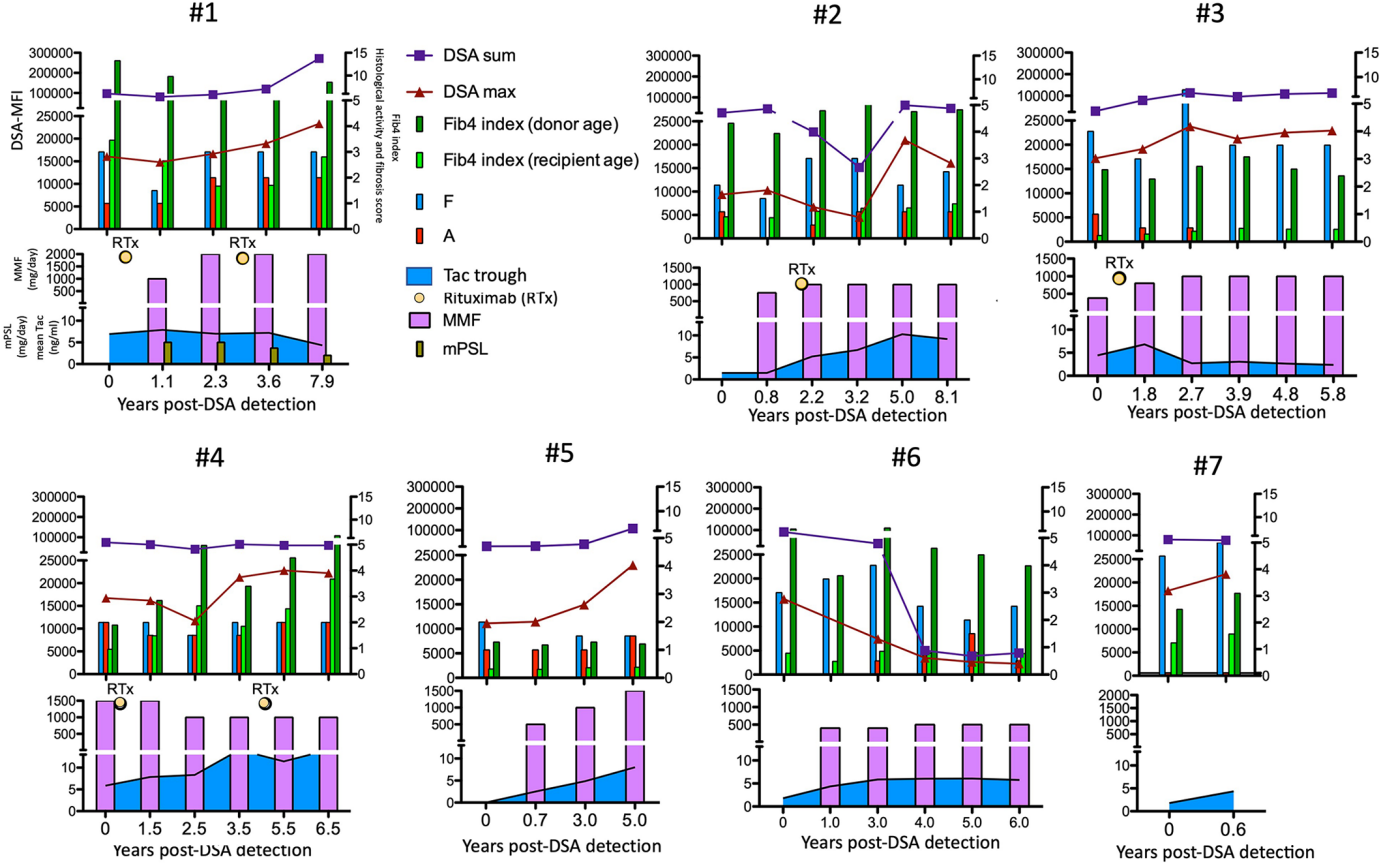
The time courses of the maximum (red triangles) and sum (blue squares) of the DSA-MFI, Fib-4 indexes calculated by ages of the recipient (fresh green bar) and donor (green bar), histological evaluations by liver biopsies (F, fibrosis score and A, METAVIR activity score) and immunosuppressants among seven patients in the high-risk group. RTx, rituximab. Tac, tacrolimus.

According to this treatment strategy, 4 of 7 subjects in the high-risk group were treated with rituximab (50 mg/kg) at 21.8 (5.2–28.5) years old (cases 1–4 in Figure 6). Rituximab immediately eliminated CD20^+^ lymphocytes from the peripheral blood of these patients. No apparent adverse effects or significant changes in the liver function, histological findings or amount of DSA were observed during the course of additional treatments (Figure 6). In addition, according to the treatment strategy for post-transplant DSA positivity shown in Figure 5, some subjects with a high DSA-MFI and low fibrosis (*n* = 11) were treated with additional tacrolimus and MMF for 2.15 ± 1.04 years, usually due to nonsignificant but slight fibrotic changes with a liver graft score of F1 (left column in Figure 4). Others with a high DSA-MFI and low fibrosis (*n* = 6) were observed without additional immunosuppressants for 2.58 ± 1.51 years (right column in Figure 4). Irrespective of additional treatments, no significant differences were observed in the patients with a high DSA-MFI and low fibrosis (Figure 4).

## Discussion

In the present study, we conducted a retrospective single-center chart review of pediatric recipients who underwent LDLT between December 1995 and November 2019. We demonstrated that 52.9% of pediatric patients developed DSA post-LDLT. A recent cross-sectional study showed that, among 157 stable pediatric LT recipients, 80 (55.6%) developed class II DSA. Of these, 37 (47.2%) showed a maximum DSA-MFI > 20,000 (20). In addition, *de novo* DSAs were detected in 60.3%–63% of pediatric LT patients (23, 24). In LDLT, 32% of pediatric patients were found to have class II DSA post-transplantation (25). Thus, approximately half of LDLT pediatric recipients developed DSA in our study, which was comparable to the findings of previous studies.

Regarding the MFI threshold of DSA, no clear consensus has been established (26). A study in kidney showed that preformed DSA with a maximum MFI of >4,900 can predict an occurrence of antibody-mediated rejection with a sensitivity of 85.7% and a specificity of 73.1% (27). Induction therapy including thymoglobulin and intravenous immunoglobulin therapy allowed patients with a DSA-MFI < 5,000 to undergo successful kidney transplantation. In addition, post-transplant DSA with an MFI > 3,000–6,000 was reportedly associated with a poor graft outcome in kidney transplantation (6). The candidate cut-off value for the DSA-MFI with an increased risk of kidney transplantation was therefore set at approximately 5,000.

Class I and II post-transplant DSA with an MFI ≥ 5,000 has been reportedly associated with an increased risk of fibrosis progression in HCV-viremic LT patients (11) as well as ABO-compatible LDLT patients (28). In addition, DSAs with an MFI > 5,000 were significantly correlated with a poor graft outcome in a large cohort study of 749 LT patients (8). Another study demonstrated that C3d-binding DSA but not only DSA with an MFI > 10,000 was significantly associated with a poor LT graft outcome (29). Furthermore, a cross-sectional study of 157 pediatric LT patients demonstrated that a class II DSA-MFI sum >20,000 was associated with an increased risk of graft fibrosis, portal inflammation and elevated C4d scores (20). Our study showed that graft fibrosis progression (≥2) was found among patients with a maximum DSA-MFI ≥ 9,300 and a DSA-MFI sum ≥25,000. Although the DSA-MFI does not directly indicate the quantity of DSA, the DSA-MFI was correlated with titer values until reaching 10,000 (30). Further studies will be needed in order to precisely discriminate the information of harmful DSA by not only a large amount of DSAs but IgG subtypes, complement biding and antigen-antibody dissociation.

We also found a certain number of subjects without graft fibrosis progression despite having an elevated titer of DSA. We therefore clearly discriminated the groups by cut-off of DSA-MFI and graft fibrosis progression (Figure 3). A previous study showed that it was not necessary to exclude pediatric LT patients developing *de novo* DSA from inclusion criteria for a weaning immunosuppression protocol (14). Those authors found that 15 (47%) subjects with *de novo* DSA, including 4 with a maximum MFI > 20,000, achieved complete withdrawal of immunosuppression despite being less likely to be operationally tolerant (14). Furthermore, protocol liver biopsies revealed normal histological features among some subjects developing *de novo* DSA (23). Thus, the clinical outcome in the presence of *de novo* DSA may depend on additional risk factors, such as DSA factors (e.g., complement binding, IgG subtype), recipient immunological factors (e.g., background immune disease, rejection episodes) and graft factors (e.g., size, ages, cold ischemia time) (31).

A previous study demonstrated that the combined presence of DSA and C4d positivity was associated with histological features of fibrosis and portal inflammation in long-term pediatric LT recipients (9, 32). In addition, *de novo* DSA in pediatric LT was associated with both inflammation and fibrosis as well as a significant increase in the number of CD8^+^ T cells in the graft (23). A study using protocol biopsies for stable pediatric LT patients identified non-biliary atresia as well as *de novo* DSA as a clinical risk factor for histological graft injuries (20). In the present study, all three DSA-positive patients who underwent LT for fulminant hepatitis displayed graft fibrosis progression (Table 2). In addition, two of these patients (#1 and #4 in Figure 6) were eventually registered on a waiting list for deceased donor LT due to cirrhotic changes despite extensive immunosuppressant administration, suggesting that recipient immunological characteristics may affect the graft outcome.

Furthermore, in our study, graft age was identified as a risk factor for predicting graft fibrosis. The donor age (>40 years old) was reported to be a risk factor for allograft fibrosis at 5 years post-pediatric LT (33). However, donor age was not always recognized as a risk factor for a poor graft outcome (29). Of note, we demonstrated in the present study that the graft age but not the donor age was significantly higher in the high-risk group than other groups (Table 2). As the Fib4-index calculated using age has been recognized as a useful predictor of liver fibrosis, age is absolutely an important risk factor for graft fibrosis progression. Furthermore, reportedly livers from younger donors facilitated operational tolerance in pediatric LT recipients than elderly donors (34). Thus, the actual hepatic age of the LT recipient should be considered when evaluating risk factors for graft fibrosis progression. Pediatric LDLT patients occasionally received a living graft from grandparents, as shown in the high-risk group in Table 2. In the presence of DSA with a high MFI, patients with additional risk factor should be carefully evaluated and monitored for graft fibrosis progression.

The treatment protocol for *de novo* DSA-positive patients has been applied since 2015 in our institute (Figure 5). Although it was difficult to decide on the additional immunosuppressants requirement, the development of *de novo* DSAs due to immune responses to donor antigens may indicate insufficient immunosuppression. The consensus regarding managing modifiable risks in the transplantation group states that, in cases with a DSA-MFI of ≥5,000 and histopathological chronic antibody-mediated rejection, an increased dose of calcineurin inhibitors and the introduction of MMF and other immunosuppressants are recommended (35). When histopathological findings show involvement of hepatitis, similar to autoimmune hepatitis, adding steroid treatment may be recommended (35). In addition, a scoring system based on histological findings may help determine the most appropriate treatment strategy (36). A score of <13 of chronic antibody-mediated rejection scoring system indicates no need for additional treatment, while a score of >27.5 predicts a 50% chance of graft failure, leading to the need for additional immunosuppressant administration (37).

We observed no fibrosis progression in the low-risk group for several years. However, we did encounter a subject with a high DSA-MFI and subtle fibrotic changes who was treated with an increased dosage of calcineurin inhibitor and additional MMF. This patient developed diffuse large B cell lymphoma as a post-transplant lymphoproliferative disease requiring chemotherapy. Thus, careful decision making to ensure optimal immunosuppression and close follow-up are required.

Of note, we were unable to conclude the requirements for additional treatment even for the high-risk group in the present study. Further investigations based on accumulating evidence and in-depth analyses, i.e., molecular profiling, may provide useful information to help decide on additional and proper immunosuppression. In addition, our study is limited by its single-institute setting. To precisely determine the cut-off DSA-MFI value for predicting graft fibrosis, validation using multicenter data will be needed.

In conclusion, a high DSA-MFI of ≥10,000 predicted graft fibrosis progression in pediatric LT. Patients with additional risk factors for graft fibrosis progression, such as an older graft age, higher Fib4 index and lower platelet count, should be considered for a histological evaluation. The need for additional immunosuppressants in high-risk pediatric LT patients warrants a further examination.

## Data Availability

The original contributions presented in the study are included in the article/**Supplementary Material**, further inquiries can be directed to the corresponding author.
