# Supplementary material for: Post-transplant donor-specific anti-HLA antibodies with a higher mean fluorescence intensity are associated with graft fibrosis in pediatric living donor liver transplantation
Source: Front Pediatr. 2023 Apr 25;11:1172516. doi: 10.3389/fped.2023.1172516 (PMC10168538; doi:10.3389/fped.2023.1172516)
Supplement: Supplementary file 1 [file Image1.pdf]

Supplementary Figure 1

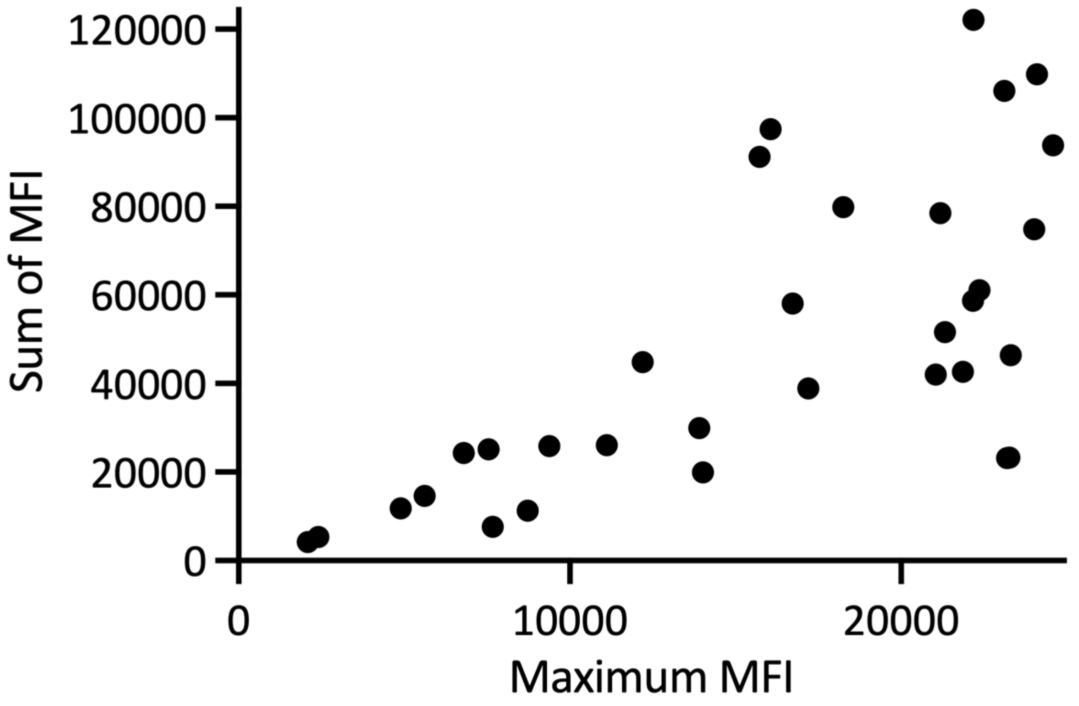

Supplementary Fig. 1. The correlation between the sum and maximum of DSA-MFI in the pediatric LT patients.
